# Intersubject Variability in Cerebrovascular Hemodynamics and Systemic Physiology during a Verbal Fluency Task under Colored Light Exposure: Clustering of Subjects by Unsupervised Machine Learning

**DOI:** 10.3390/brainsci12111449

**Published:** 2022-10-27

**Authors:** Hamoon Zohdi, Luciano Natale, Felix Scholkmann, Ursula Wolf

**Affiliations:** 1 Institute of Complementary and Integrative Medicine, University of Bern, 3012 Bern, Switzerland; 2 Biomedical Optics Research Laboratory, Neonatology Research, Department of Neonatology, University Hospital Zurich, University of Zurich, 8091 Zurich, Switzerland

**Keywords:** functional near-infrared spectroscopy, fNIRS, systemic physiology augmented functional near-infrared spectroscopy, SPA-fNIRS, colored light exposure, verbal fluency task, CLE-VFT, unsupervised machine learning, *k*-means clustering

## Abstract

There is large intersubject variability in cerebrovascular hemodynamic and systemic physiological responses induced by a verbal fluency task (VFT) under colored light exposure (CLE). We hypothesized that machine learning would enable us to classify the response patterns and provide new insights into the common response patterns between subjects. In total, 32 healthy subjects (15 men and 17 women, age: 25.5 ± 4.3 years) were exposed to two different light colors (red vs. blue) in a randomized cross-over study design for 9 min while performing a VFT. We used the systemic physiology augmented functional near-infrared spectroscopy (SPA-fNIRS) approach to measure cerebrovascular hemodynamics and oxygenation at the prefrontal cortex (PFC) and visual cortex (VC) concurrently with systemic physiological parameters. We found that subjects were suitably classified by unsupervised machine learning into different groups according to the changes in the following parameters: end-tidal carbon dioxide, arterial oxygen saturation, skin conductance, oxygenated hemoglobin in the VC, and deoxygenated hemoglobin in the PFC. With hard clustering methods, three and five different groups of subjects were found for the blue and red light exposure, respectively. Our results highlight the fact that humans show specific reactivity types to the CLE-VFT experimental paradigm.

## 1. Introduction

Color and colored light have always fascinated humans and played a vital role in daily life. In our modern society, we are increasingly exposed to various colored light sources, ranging from illuminated advertising boards to computer screens, room lighting, and smartphones. In addition, colors and colored lights can also be parts of different learning environments. It has been demonstrated that colored lights (or colors) have significant effects on students, influencing their emotions, mood, performance, and systemic physiology [1,2,3,4]. Selecting an optimal learning environment with a specific colored light may enhance cognitive performance in the context of education. In spite of the broad range of colored light applications, the influence of colored light on human physiology is still rarely studied and is therefore of substantial interest for science and society.

Systemic physiology augmented functional near-infrared spectroscopy (SPA-fNIRS) is a powerful approach to study the physiological state of a human and body–brain interactions [5]. This approach can also be used to understand how the entire body reacts to stimulus/task paradigms [5]. In our previous studies, we employed this approach to investigate the visual and non-visual effects of colored light exposure (CLE) in humans [6,7,8,9]. In one of our latest studies [9], we investigated a mixed-effect of CLE and a verbal fluency task (VFT; one of the most widely applied tests for the assessment of cognitive function [10,11,12]) by SPA-fNIRS. In this study [9], subjects were *manually* classified into different groups based on their hemodynamic response patterns of oxygenated hemoglobin ([O_2_Hb]) in the prefrontal (PFC) and visual cortex (VC). Seven and five different hemodynamic patterns were found for red and blue light exposure, respectively. The *manual* classification of subjects was the very first step in analyzing such data, providing valuable information about individual differences in hemodynamic responses during a CLE-VFT. However, the impact of systemic physiology was not directly taken into account for such a classification. In fact, considering several parameters, i.e., cerebrovascular and systemic physiological parameters, the *manual* classification of subjects is complex and not practicable. In the present study, we aimed to extend this approach by using an *automatic* classification method. More parameters have been considered in the process of *automatic* classification, which is expected to provide a better overview and understanding of different subjects’ reactions.

Machine learning (ML) algorithms can be used to extract new information from physiological data. Such algorithms can help in the *automatic* classification of different subjects’ reactions due to a CLE-VFT. Unsupervised ML is a type of algorithm for learning from unlabeled data, which aims to find patterns or intrinsic structures from input features, facilitating flexible, general, and *automated* methods of ML [13,14]. Clustering analyses, a subsection of unsupervised ML, partition input data into distinct groups based on similarities between observations [15,16].

Using unsupervised ML, we aimed to investigate whether the CLE-VFT causes different subjects’ reactions in cerebrovascular hemodynamics and systemic physiology measured with SPA-fNIRS. Particularly, we analyzed the performance of a variety of different clustering methods. We hypothesized that unsupervised ML would enable the classification of the response patterns, which would provide new insights into the common response patterns between subjects.

## 2. Materials and Methods

### 2.1. Subjects

In total, 32 healthy right-handed adults were recruited in this study (15 men and 17 women, age: 25.5 ± 4.3 years). The size of the sample was calculated with a power analysis to detect substantial effects (effect size: *d* = 0.59; based on our previous study results investigating the different physiological effects of light exposure with two colors) at a *p* < 0.05 and a power of >0.8. Subjects were all well-educated (i.e., university students or individuals with university degrees), non-smokers, medication-free, and without any significant history of neurological, cardiovascular, or respiratory disease. To avoid physiological changes due to confounders, subjects were asked to refrain from consuming stimulants (e.g., coffee, tea, or energy drinks) and from eating for two hours before the measurements. The study had a quantitative, cross-over, randomized, and semi-blinded design. All measurements were conducted at the Institute of Complementary and Integrative Medicine of the University of Bern. The recruitment and data collection were performed over a time span of 15 months (August 2017–October 2018). Recruitment was conducted via the distribution of flyers and an online advertisement on the University of Bern website as well as inquiries from colleagues, friends, and acquaintances. Subjects were financially compensated for their participation.

### 2.2. Experimental Protocol and Measurement Setup

Each subject was exposed to two colored lights (red and blue) for 9 min on two different days but at the same time of day (± half an hour). The color order was randomized, and the light illuminance was set at 120 lux for each color. Subjects sat comfortably in a reclining chair and were asked to perform a VFT while they were exposed to the colored light. Before and after the CLE-VFT, subjects were in a rest phase (darkness, without any task) for 8 min and 15 min, respectively. The subjects performed the experiment with their eyes open for both resting sessions as well as the CLE-VFT phase. Subjects produced 58 ± 12 (mean ± SD) correct nouns during the red light exposure and 57 ± 15 during blue light exposure. No significant difference in the VFT performance was found between the red and blue light exposure.

The SPA-fNIRS approach comprised a multi-channel frequency-domain near-infrared spectroscopy (FD-NIRS) device (Imagent, ISS Inc, Champaign, IL, USA) and three devices to measure systemic physiological parameters. FD-NIRS is able to determine the absolute values of [O_2_Hb], deoxygenated hemoglobin ([HHb]), total hemoglobin ([tHb]), and tissue oxygen saturation (StO_2_) in the PFC and VC. Our fNIRS was sensitive to the brain and, as a multi-distance system, minimized the impact of extracerebral blood flow changes on the data [17,18,19,20]. Heart rate (HR), mean arterial blood pressure (MAP), and arterial oxygen saturation (SpO_2_) were measured by a SOMNOtouch NIBP device (SOMNOmedics GmbH, Randersacker, Germany). A NONIN LifeSense (NONIN Medical, Plymouth, MN, USA) device was employed to record the respiration rate (RR) and the end-tidal carbon dioxide (P_ET_CO_2_). Skin conductance (SC) was determined with a VERIM system (Mind-Reflection, Tallinn, Estonia). All data were recorded simultaneously.

A more detailed description of the experimental protocol, as well as the SPA-fNIRS setup, can be found in our previous studies [8,9].

The planning of the study, the data analysis, and the reporting of the results were conducted according to recently published fNIRS guidelines [21].

### 2.3. Signal Processing and Machine Learning

To avoid the effects of confounders, a homogenous sample was selected for data analysis. All subjects were Swiss German speakers. To have a sample in a small age range (20 to 30 years), two subjects over 30 years old were excluded from the analysis. By removing these two subjects, we avoided the need to correct for age when performing the statistical analysis. Signal preprocessing was performed according to our previous study [9]. For each time-dependent parameter, the area under the curve (AUC) of the CLE-VFT phase was calculated for each subject in both conditions (red and blue). In total, 15 different parameters (features) were investigated: [O_2_Hb], [HHb], [tHb], and StO_2_ in the PFC and VC as well as HR, MAP, SpO_2_, RR, P_ET_CO_2_, SC, and task performance. Subsequently, the features were subjected to min–max normalization in order to bring all of them to a comparable scale. Min–max normalization has the advantage of exactly preserving all relationships in the data [22,23]. A principal component analysis (PCA) was applied in order to reduce the dimensionality of the feature space so that unsupervised ML algorithms could be implemented effectively. The observations were projected on the *n* first principal components of the data, and data in the projected space were used for unsupervised ML. The number of selected principal components was such that around 80% of the variance was kept. This allowed us to significantly reduce the dimensions of the data while not losing much information. It has been demonstrated that for descriptive purposes at least 80% of the variance should be explained by the principal components. In this study, two dimensions were sufficient to account for >80% of the variance in the data, while the variance contributed by other principal components was small [24,25].

The aim of the next step of the data analysis was to select a maximum of five features among the 15 features investigated in this study so that one was chosen from the PFC parameters, one was chosen from the VC, and three were chosen from the systemic physiology. Since all of our subjects were students with almost equal verbal fluency skills, in unsupervised ML the task performance of the subjects had a low and insignificant impact on the classification of subjects. Using an in-house-developed algorithm, all possible combinations of the features were found and scored (silhouette value). Then, all combinations with silhouette values above 0.6, which indicates a good clustering (i.e., clusters are well-separated) [26,27,28], for a range of cluster numbers (*k* = 2 to *k* = 9) were selected for both the blue and red light conditions, and the best common combinations in both conditions were identified. Finally, using unsupervised ML, a variety of different clustering methods were used to classify subjects. These methods included *k*-means, *k*-medoids, hierarchical clustering, a Gaussian mixture model (GMM), the density-based spatial clustering of applications with noise (DBSCAN), and clustering via self-organizing maps (SOM). Three clustering criteria were used, including the silhouette score [29], the Calinski–Harabasz index [30], and the Davies–Bouldin index [31], in order to evaluate the quality of clustering and the performance [32].

Signal processing steps and machine learning were performed in MATLAB (R2021b, MathWorks, Inc., Natick, MA, USA).

## 3. Results

Using *k*-means clustering as a method and the silhouette index as a clustering criterion, the five best combinations of the cerebrovascular and systemic physiological parameters were detected for subject classification (Table 1). SC was the most important and sensitive parameter for the clustering, as observed in all five sets, while SpO_2_ also played a significant role in subject classification. SpO_2_ and SC were the common components of the first three categories, together with other parameters, leading to three and five different groups of subjects for the blue and red light exposure, respectively. Moreover, as Table 1 shows, [O_2_Hb] and [HHb] had greater impacts on the classification of subjects compared to the other two cerebrovascular parameters (i.e., [tHb] and StO_2_). With these five sets of parameters, subjects were suitably classified into different groups. The best subject classification was obtained according to the changes in the following parameters: [HHb] in the PFC, [O_2_Hb] in the VC, P_ET_CO_2_, SC, and SpO_2_.

For the next data analysis step, the best subject classification set was chosen, and several clustering methods were employed to determine the optimal number of clusters within each condition. Table 2 shows the best number of groups for all clustering algorithms utilized in this study. In almost all methods, two out of three clustering criteria (bold numbers) confirmed that the subjects were classified into three and five groups for the blue and red light exposure, respectively. Moreover, hard clustering algorithms (*k*-means, *k*-medoids, and SOM) had higher clustering performance (blue: silhouette index = 0.77, red: silhouette index = 0.88) compared to the soft clustering algorithm (GMM; blue: silhouette index = 0.71, red: silhouette index = 0.53) and the two other methods (hierarchical clustering; blue: silhouette index = 0.63, red: silhouette index = 0.87 and DBSCAN; blue: silhouette index = 0.2, red: silhouette index = 0.87).

**Figure 1:**
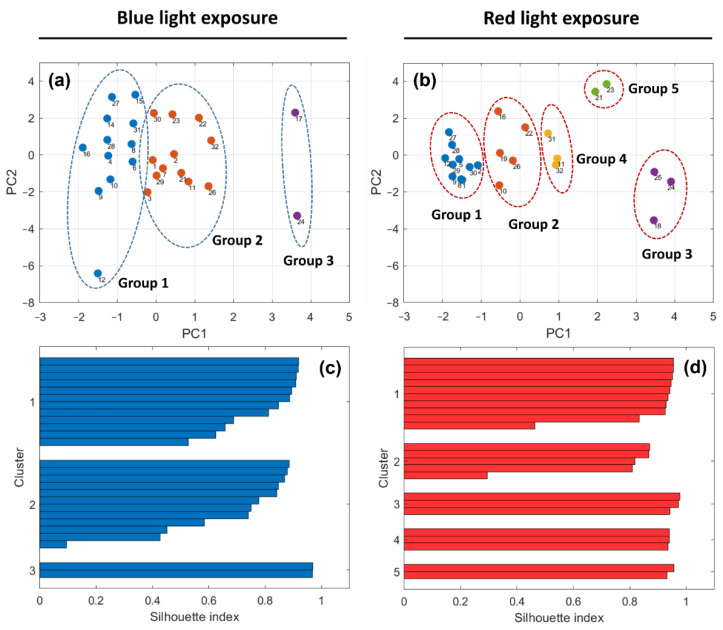
Classification of subjects based on changes in [HHb]-PFC, [O_2_Hb]-VC, P_ET_CO_2_, SC, and SpO_2_ using k-means clustering: (**a**,**b**) PCA scores and (**c**,**d**) silhouette values of each individual for the blue and red light exposure. The numbers next to the data points indicate the subjects’ identification codes.

Considering the best set of parameters ([HHb]-PFC, [O_2_Hb]-VC, P_ET_CO_2_, SC, and SpO_2_), Figure 1 visualizes the PCA scores and silhouette index values, obtained by *k*-means clustering, of each subject for the blue and red light exposure conditions. Three and five different groups of subjects were found for the blue (silhouette index = 0.77) and red (silhouette index = 0.88) light exposure, respectively. At the individual level, the silhouette plots show that most subjects have a considerable silhouette index value (i.e., >0.6), indicating that the group is relatively well-separated from neighboring groups. In 81% of the subjects, the silhouette index value was >0.6 for the blue light exposure condition, whereas 91% were >0.6 for the red light exposure. This specifies that subjects were well-separated (with distinctive reactions) during the red light exposure compared to blue light exposure. Figure 1a,b depict that some subjects reacted the same, i.e., relatively similar PCA scores, to both colored lights (e.g., subjects #9, #22, #24, and #28) and some others showed completely different reactions (e.g., subjects #16, #21, #23, and #31) to blue and red lights, i.e., dissimilar PCA scores. Considering the first group for both light colors, subjects exposed to blue light showed more scattered reactions than during red light exposure. To some extent, this was also observed in the other groups, as the second group in the blue light condition was relatively equivalent to the integration of the second and fourth groups in red light.

## 4. Discussion

### 4.1. Red Light Causes Greater Intersubject Variability in the Physiological Reactions Compared to Blue Light Exposure

In this study, we found that during the CLE-VFT subjects exposed to red light were classified in more groups than those exposed to blue light. This finding aligns with our previous study results, where subjects were manually classified into different groups [9]. A relatively higher number of clusters in red compared to blue implies that red light may cause broader and more varied effects on human physiology compared to blue light. Red is equally associated with both positive (e.g., happiness, joy, and excitement) and negative (e.g., anger, danger, and fear) emotional perceptions, while blue is more associated with positive concepts (e.g., calm, comfort, and contentment) [33,34,35,36,37]. Red, in general, has been known as a unique and special color, and people who like this color are supposed to be active, influential, cheerful, competitive, optimistic, and action-oriented [38,39]. It has been hypothesized that red has negative meanings (failure) and aversive implications (avoidance motivation) in achievement contexts. On the other hand, it also carries positive, appetitive meanings and facilitates approach-relevant responding in relational contexts [39]. Our results imply that red light might lead to various positive and negative reactions in the subjects. These reactions, which were detected by unsupervised ML based on changes in SPA-fNIRS parameters during the CLE-VFT, can be classified into five categories, whereas blue light caused fewer clusters with more dispersed reactions, as blue appeals to almost everyone and carries more general and limited concepts.

### 4.2. Hard Clustering Methods Have Better Clustering Performance

In the current study, we utilized six different clustering methods in order to explore a wide range of possible solutions for identifying different subjects’ reactions during the CLE-VFT. The optimal number of clusters was determined using three criteria: the silhouette index, the Calinski–Harabasz index, and the Davies–Bouldin index. Using these criteria, the optimal numbers of clusters were generally equal to three and five for the blue and red light exposure, respectively. However, for the DBSCAN method, in the blue light condition the optimal number of clusters was equal to two. DBSCAN is known to be robust to noise points and can exclude data points from being part of any group, which decreases the impacts of outliers on its classification performance [40,41]. It seems that this method sorted two data points from the blue light (#17 and #24) into a separate “noise” cluster since they were too dissimilar to the rest of the dataset. Therefore, this algorithm found “two” to be the optimal number of clusters, which is not necessarily true in our case because we believe that these two data points are not real outliers but two different reactions of subjects to the blue light, which incidentally both belong to the same group (group 3, approved by other unsupervised ML methods). Using a larger number of subjects in future studies would enable the determination of whether such data points are outliers or specific reactions to the blue light. Moreover, we demonstrated that hard clustering algorithms (*k*-means, *k*-medoids, and SOM) have a higher clustering performance compared to the soft clustering algorithm (GMM) and the two other methods (hierarchical clustering and DBSCAN). In hard clustering methods, each data point either belongs to a cluster completely or not, while soft clustering methods are more flexible and can assign a data point to more than one cluster. *k*-means clustering is a simple, powerful, and widely used approach for classification, aiming to classify *n* observations into *k* clusters where each observation belongs to the cluster with the nearest mean [42,43]. *k*-medoids is similar to *k*-means, but instead of taking the mean value of the data points in a cluster, the most centrally located data point is considered as the reference point [14,44]. SOM is a special class of neural network based on competitive learning, which transforms a dataset into a topology-preserving 2D discrete map [13,14]. By employing all unsupervised ML methods, we found that the clustering performances of *k*-means, *k*-medoids, and SOM were the same and were the highest among all other methods for both the red and blue light conditions. Therefore, we can conclude that hard clustering methods suit this type of physiological data best. Compared to the hierarchical clustering method, *k*-means and *k*-medoids recover more stable clusters, classify messy high-dimensional data more accurately, and have less computational complexity [14,43,45]. Mangiameli et al. also demonstrated the superior accuracy and robustness of SOM in comparison with hierarchical clustering methods [46]. GMM, a fuzzy or soft clustering method, uses partition-based clustering where data points come from different multi-variate normal distributions with certain probabilities [47]. In line with our findings, Maaoui and Pruski found that *k*-means and SOM perform better than GMM when clustering physiological signals [48]. Finally, it has frequently been observed (Table 2) that the optimal number of groups evaluated by the Calinski–Harabasz index is higher than for the two other criteria, especially for hard clustering algorithms. This could mainly be attributed to the major drawback of this criterion, which is generally higher for convex globular clusters, namely *k*-means [49].

### 4.3. Changes in Systemic Physiological Activity Help to Classify the Individual Physiological Responses to a Task/Stimulation

As the traditional method for data classification, *manual* classification can be biased and inconsistent but not necessarily worse or less accurate than *automatic* classification. The data used in the current study had also been analyzed in our previous research [9] where subjects were manually classified into different groups based only on their hemodynamic response patterns of [O_2_Hb] in the PFC and VC. Although the subject-specific analysis and data classification were successfully performed in the previous study [9], the effects of the systemic physiological parameters on subject clustering were not directly taken into account. Therefore, an *automatic* classification was needed to provide a meaningful and better classification that considered both the cerebrovascular and systemic physiological data. In the current study, we investigated using unsupervised ML with the SPA-fNIRS approach to find different subjects’ reactions based on changes in both cerebrovascular hemodynamics and systemic physiology. The *manual* classification of subjects with fNIRS signals was the very first step in analyzing such data, providing valuable information about individual differences in cerebrovascular hemodynamics during a CLE-VFT. However, a reliable and accurate interpretation of the changes in the fNIRS signals and thus a better subject classification cannot be obtained without considering systemic physiological parameters. In terms of classification, taking five features (cerebrovascular and systemic physiological parameters) instead of only two features (cerebrovascular parameters) into account was the main strength of this study compared to the previous one. It definitely facilitates a better understanding of the role and influence of each individual in a cluster. For example, considering the first group of the red exposure, the range of changes in SC during the CLE-VFT was from 0.03 to 1.78 µS, while highly significant positive SC changes were observed for the third cluster (ΔSC > 11 µS). [O_2_Hb] changes in the VC for the individuals of the first cluster were in the range of positive to insignificant (0.66 ± 1.05 µM), but they were significantly lower for the third group (−0.83 ± 0.29 µM) compared to the first group. In addition, the changes in [HHb] in the PFC for most subjects in the first group were significantly negative, while they were insignificant for the subjects in the third group. Individuals in the first and third groups showed a wide range of changes in P_ET_CO_2_ and SpO_2_ during the CLE-VFT. In other words, no specific common P_ET_CO_2_ and SpO_2_ patterns were found for these two groups. The findings under the blue light condition were roughly in line with the red light results (first cluster: −0.11 µS < ΔSC < 2.33 µS, Δ[O_2_Hb]-VC: 1.28 ± 1.23 µM; third cluster: ΔSC > 11.4 µS, Δ[O_2_Hb]-VC: 0.55 ± 0.37 µM). Unsupervised ML enabled us to provide a better overview and understanding of different subjects’ reactions based on not only the fNIRS signals but also systemic physiology. In our previous study [9], the effect of systemic physiology was not directly taken into account for the classification of subjects due to some limitations of the *manual* method. However, the current study shows that more parameters can be taken into consideration for subject classification with unsupervised ML. It was also found that one of these systemic physiological parameters, i.e., SC, along with [O_2_Hb]-VC play the most important roles in the clustering since the individuals of each group had special characteristics for these two parameters. SC reflects the state and activity of the autonomic nervous system. The various forms of SC changes observed among all individuals were associated with the stress that subjects experienced during the VFT task. In other words, subjects experienced different stress levels while performing the task, which was mostly identified by measuring electrodermal activity. On the other hand, P_ET_CO_2_ and SpO_2_ were found to be less sensitive parameters for the clustering. Such unique findings cannot be obtained except by an *automated* method (e.g., unsupervised ML). Moreover, it seems that statistically significant correlations between some cerebrovascular and systemic physiological parameters play a vital role in subject classification. Considering the systemic physiological parameters of the first two categories (P_ET_CO_2_, SC, SpO_2_, and MAP), statistically significant correlations were found as follows: (i) blue light exposure: [O_2_Hb]-VC vs. SC (*r* = 0.48, *p* = 0.008), [O_2_Hb]-VC vs. P_ET_CO_2_ (*r* = −0.28, *p* = 0.014), and [O_2_Hb]-PFC vs. MAP (*r* = 0.56, *p* = 0.002); (ii) red light exposure: [HHb]-PFC vs. SpO_2_ (*r* = −0.44, *p* = 0.015), [O_2_Hb]-VC vs. P_ET_CO_2_ (*r* = −0.4, *p* = 0.035), [O_2_Hb]-VC vs. SpO_2_ (*r* = 0.61, *p* < 0.001). In our previous studies, we also showed that changes in P_ET_CO_2_ have strong effects on cerebrovascular hemodynamics [50,51]. The correlation of SC and cerebrovascular hemodynamics has been reported elsewhere [52,53,54,55,56]. It has also been shown that MAP and SpO_2_ correlate with the changes in the fNIRS signals in the PFC and the motor cortex [9,57]. Caldwell et al. designed a model providing valuable information regarding the possible confounding factors of fNIRS measurements [58], showing that depending on the degree of the changes in P_ET_CO_2_ and MAP, specific hemodynamic responses are induced.

### 4.4. Limitations

The current study has the following limitations: (1) Although the number of participants was calculated with a power analysis, a more optimal number of groups with more subjects in each cluster might have been found if the number of participants had been larger. (2) The fNIRS measurement setup did not cover the entire head, and therefore the whole brain was not measured. (3) Different cognitive tasks might affect subjects differently, depending on the nature of the task. As a very common example, females generally perform better than males in the VFT, finger tapping, and item memory, while males generally perform better in visual-spatial tasks such as mathematical tasks and mental rotation [1,59]. Moreover, non-mathematical tasks activate the whole PFC and the parietal cortex, supporting more general cognitive operations (e.g., attention and emotion) rather than specific modules for calculation, while mathematical processing may recruit only the left frontal cortex and the intraparietal sulcus [60,61,62]. Thus, our results cannot be transferred to other cognitive tasks. However, this will be investigated in future studies in combination with CLE. (4) While red and blue are the most common and widely used colored lights in science and society, other colors should also be investigated with our experimental paradigm and SPA-fNIRS approach.

## 5. Conclusions

For the first time, we used machine learning to investigate the intersubject variability in hemodynamic and systemic physiological responses due to a VFT under CLE. Since the manual classification of subjects with several parameters is complex and, in most cases, not practical, machine learning is an alternative method to categorize subjects into different groups. Based on the SPA-fNIRS parameters, we suitably classified the subjects into different groups using unsupervised machine learning in which the number of groups was different between the red and the blue light exposure. We showed that SC and [O_2_Hb]-VC play vital roles for the clustering, as the same response patterns between most subjects in each group were found for these two parameters. Since each individual reacts differently to the CLE, it would be advantageous to generate an algorithm that enables us to understand how each individual responds to the CLE based on cerebrovascular and systemic physiological changes. The newly applied data analysis is the very first step of designing an algorithm to assist in determining who reacts in a specific physiological way to a colored light exposure.

## Figures and Tables

**Table 1:** Summary of the best five sets of cerebrovascular and systemic physiological parameters for subject classification. The optimal number of clusters with the corresponding silhouette index criterion is shown for both the blue and red light exposure conditions.

Features	Optimal Number of Clusters (Blue Light Exposure)	Silhouette Index (Blue Light Exposure)	Optimal Number of Clusters (Red Light Exposure)	Silhouette Index (Red Light Exposure)
[HHb]-PFC, [O_2_Hb]-VC, P_ET_CO_2_, SC, SpO_2_	3	0.77	5	0.88
[O_2_Hb]-PFC, [HHb]-VC, SC, MAP, SpO_2_	3	0.75	5	0.87
[HHb]-PFC, [HHb]-VC, RR, SC, SpO_2_	3	0.76	5	0.85
[HHb]-PFC, [HHb]-VC, P_ET_CO_2_, SC, HR	3	0.72	7	0.86
[O_2_Hb]-PFC, [O_2_Hb]-VC, SC, HR, MAP	4	0.70	6	0.87

**Table 2:** The optimal number of clusters for the best set of parameters ([HHb]-PFC, [O_2_Hb]-VC, P_ET_CO_2_, SC, and SpO_2_) using six different clustering methods and evaluated with three clustering criteria.

Condition	Clustering Criteria	*k*-Means	*k*-Medoids	Hierarchical Clustering	GMM	SOM	DBSCAN
Blue light exposure	Silhouette index	**3**	**3**	**3**	2	**3**	2
	Calinski–Harabasz index	7	7	**3**	**3**	6	8
	Davies–Bouldin index	**3**	**3**	2	**3**	**3**	2
Red light exposure	Silhouette index	**5**	**5**	**5**	4	**5**	**5**
	Calinski–Harabasz index	7	7	**5**	**5**	7	**5**
	Davies–Bouldin index	**5**	**5**	4	**5**	**5**	9

## Data Availability

The data that support the findings of this study are available from the corresponding author upon reasonable request.
